# Supplementary material for: Medication Adherence in Medicare-Enrolled Older Adults with Chronic Obstructive Pulmonary Disease before and during the COVID-19 Pandemic
Source: J Clin Med. 2022 Nov 26;11(23):6985. doi: 10.3390/jcm11236985 (PMC9741303; doi:10.3390/jcm11236985)
Supplement: Supplementary file 1 [file jcm-11-06985-s001.zip › jcm-2012861-supplementary.pdf]

Supplementary information

Table S1. Medication included in each medication category .

Medication category	Medications
SABA	Albuterol (salbutamol), Levalbuterol, Terbutaline, Fenoterol
SAMA	Ipratropium, Oxitropium
SAMA-SABA	Ipratropium-Fenoterol, Ipratropium-Salbutamol
LABA	Arformoterol, Formoterol, Salmeterol, Olodaterol, Indacaterol
LAMA	Aclidinium, Glycopyrrolate,Tiotropium, Umeclidinium, Glycopyrrolate, Revefenacin
LAMA-LABA	Aclidinium/formoterol, Glycopyrrolate/indacaterol, Glycopyrrolate /formoterol, Tiotropium/olodaterol, Umeclidinium/vilanterol
LABA-ICS	Budesonide-formoterol, Fluticasone propionate-salmeterol, Fluticasone propionate-salmeterol, Fluticasone furoate-vilanterol, Mometasone-formoterol
LAMA-LABA-ICS	Budesonide/glycopyrrolate/formoterol, Fluticasone furoate/umeclidinium/vilanterol, Beclometasone/formoterol/glycopyrronium

Abbreviations: SABA, short-acting beta agonists; SAMA, short-acting muscarinic antagonists; LABA, long-acting β-agonists; LAMA, long-acting muscarinic antagonists; ICS, inhaled corticosteroids. .
